# Report of the Diseases of Edinburgh, for July 1808

**Published:** 1808-09

**Authors:** John Roberton

**Affiliations:** Surgeon


### *Report of the Diseases of Edinburgh, for July* 1808; *by* Mr. JOHN ROBERTON, *Surgeon.*

THE weather during the first two-thirds of this month was delightful. It was warm but not disagreeably so; and I believe, upon the whole, there has not, for many years past, in this country, been such favourable weather. About the beginning of the last third of the month, we had some thunder, followed by heavy showers of rain; and near the the end of it, we experienced some heavy dews with fogs in the evening and night, and likewise some very heavy showers of rain. The thermometer has for the most part been high, and even during the fogs and rain near the end of the month, the barometer stood high.

During this month no epidemic has existed with any degree of violence, consequently the Medical Practitioner's list has not been very crowded; and were it not for the indiscriminate, the barbarous, the ridiculous practice of at all times, but especially when business is otherwise lax, unnecessarily burning the urethra, œsophagus, rectum, &c. with caustic, the practitioner will nearly be idle. From the very great number of families also, which go into the country about this time, there is of course less sick left with us than at any other period of the year.

Ophthalmia has again, in a great measure, abated with us, as scarcely any cases of it are to be met with in practice.

The febrile complaints, which I mentioned in my last Report, while they seem to be abating in one part of the city, seem as quickly produced in another. This we must lay our account with, while those magazines of disease, from which febrile complaints in our neighbourhood arise, are suffered to exist.

Small-pox have again made their appearance among us, and in many parts of our city they, at present, exist in great profusion, and of a very bad kind. The heat of the weather too, probably increases their malignity; and it certainly certainly renders the complaint excessively disagreeable to those who are obliged to live in the same apartment with those affected by this loathsome disease. The medical attendant can do little more than attempt to remove this or any other disease, when it has been actually formed, and represent the effects of such complaints on Society, that the Legislators of the Country may cause the proper measures to be adopted for their prevention. The small pox has lately, and very justly, occupied the public mind in no small degree; we have now a sure preventive of it in our power; and a law to adopt these means ought to be made, and the observance of it strictly insisted on. If these measures, so loudly calling for legal interference, are still overlooked, we know well who ought to be blamed; and, in such omissions, we only see a part of that system of neglect, which, to the disgrace of our rulers, has too long existed in almost every thing that relates to the preservation of the health and lives of individuals, the Medical police of the country.

Bowel complaints have prevailed to a very considerable extent, probably caused by the use of unripe fruits, with which, from the nature of the climate, this country abounds; or to the immense quantities of *whiskey* drank after eating them. There is the greatest reason to believe that these substances taken into the stomach, are the cause of the present bowel complaints, from the manner in which the complaints make their attack. The stomach is first affected with uneasiness, soon after pain affects the head, the appetite fails, and then the diarrhœa commences, with, or sometimes without, vomiting.

I have almost uniformly, and very speedily, succeeded in my attempts to remove these complaints, by ordering an emetic to be taken; and a few hours after it had operated freely, a dose of physic to be administered.

*No. 4, St. James's Street.*

